# The past, present, and future of temporomandibular disorders in predoctoral curriculum: historical perspectives and what comes next

**DOI:** 10.22514/jofph.2026.003

**Published:** 2026-01-12

**Authors:** Elizabeth Hatfield, Shaiba Sandhu, Theodora Danciu, Daniel J. Clauw, Alexandre DaSilva

**Affiliations:** ^1^Department of Oral & Maxillofacial Surgery, University of Michigan, Ann Arbor, MI 48104, USA; ^2^Department of Oral Medicine, Oncology and Orofacial Pain, High Point University Workman School of Dental Medicine, High Point, NC 27262, USA; ^3^Department of Periodontics and Oral Medicine, University of Michigan, Ann Arbor, MI 48104, USA; ^4^Department of Anesthesiology, University of Michigan, Ann Arbor, MI 48104, USA; ^5^Department of Biomedical and Material Sciences, University of Michigan, Ann Arbor, MI 48104, USA

**Keywords:** Temporomandibular disorders, Temporomandibular joint, Orofacial pain, Education, Curriculum, Dental students

## Abstract

Approximately one-third of adults in the United States are estimated to suffer from temporomandibular disorders (TMD). Despite the widespread prevalence, effective diagnosis and management of TMD remains inadequate, contributing to patient frustration and a sense of stigmatization by healthcare providers. To address this gap, significant attention is being directed toward improving educational initiatives at all levels regarding TMD. This perspective aims to explore the historical development, current standards, and emerging trends in TMD-related education in the U.S. and Canada. From its early misconceptions as a disorder primarily caused by occlusal discrepancies, TMD education has evolved towards a biopsychosocial model that acknowledges the multifactorial nature of these disorders. Significant gaps persist in predoctoral dental curricula, hindering the development of effective clinical skills among students, despite advancements in diagnostic criteria, including the Research Diagnostic Criteria for TMD (RDC/TMD) and the more comprehensive Diagnostic Criteria for TMD (DC/TMD) and International Classification of Orofacial Pain (ICOP). Current standards for TMD education highlight the necessity for dentists to achieve competence in managing these disorders, yet the lack of standardization across schools remains a barrier. Integrating multidisciplinary and interprofessional education strategies into the curriculum offers a path forward, as these approaches foster collaborative practice and enhance patient management. Additionally, incorporating artificial intelligence (AI) and other innovative educational technologies holds the potential to revolutionize TMD education, enabling personalized learning and improved clinical decision-making. Addressing these educational gaps and embracing a standardized approach to TMD-related predoctoral education will equip future dental professionals with the knowledge and skills necessary to improve patient outcomes in TMD.

## 1. Introduction

Temporomandibular disorders (TMD) are common disorders of the temporomandibular joint (TMJ) and surrounding musculature associated with pain and functional limitations of the jaw. TMD is a collective term for more than 30 conditions with broad diagnostic criteria and overlapping etiologies making prevalence estimates a challenge. However, it is reported as the second most common source of musculoskeletal pain after chronic back pain, according to the National Institute of Dental and Craniofacial Research [1]. In 2021, TMD was reported in 31.1% of the United States (US) population [2], and a 2024 meta-analysis found that 26% of participants in North America reported to have TMD [3]. Despite the reported relatively high prevalence, patients struggle to find adequate care. A retrospective study found that patients with orofacial pain (OFP), including TMD, were referred up to 15 times and 50% received no attempt at disease management [4]. Most importantly, patients with TMD feel hopeless about their treatment, frustrated by limited provider knowledge, disappointed in their care and stigmatized by their providers [5, 6].

Given the high prevalence and impact of TMD, there is an urgent need for educational initiatives as limited knowledge remains a barrier for patients resulting in poor clinical care. In 2020, the National Academies of Science, Engineering and Medicine called for improved training in this discipline to alleviate patient suffering [7]. They recommended enhanced education in assessment, treatment, management, and referral. This aligns with reports that dental students have limited knowledge about OFP disorders [8]. Despite their knowledge gaps, dental graduates will likely manage TMD cases, as TMD patients frequently seek dental consultations, report pain in the muscles and TMJ as frequently as dentoalveolar pain, and are up to 20% more likely to utilize dental services than those without a TMD [9, 10]. Although dentists generally have a positive attitude towards treating TMD [11], they experience distress related to clinical TMD management, effective communication, and navigating insurance barriers [12]. Notably, improved knowledge correlates with reduced stress and difficulty [12], emphasizing the importance of early and comprehensive education in TMD.

Integrating TMD education into dental school curricula is essential. This narrative review will examine the US and Canadian historical initiatives, current standards, and emerging trends to identify best practices for predoctoral dental programs. It will explore how shifting paradigms in TMD pathophysiology, diagnosis, and management have led to the existing educational recommendations and standards. Finally, this perspective aims to assess emerging trends to anticipate future best practices for incorporating TMD into dental curricula.

## 2. Past

### 2.1 Shifting paradigms: TMD as a multifactorial disease

To fully grasp the evolution of TMD education in predoctoral curricula, a comprehensive understanding of the paradigmatic shifts in TMD etiology and resulting controversies is necessary. Although TMD was first mentioned as early as the fifth century, it wasn’t until 1887 that medical literature specifically described symptoms originating from the TMJ [13]. During that period, physicians managed painful symptoms of the TMJ with operative procedures, intended to address what were likely articular disc displacements [14]. The management of TMD in the dental field wasn’t mentioned until 1918 and 1934 when Prentiss and Costen, respectively, drew attention to the dental components of TMD. Prentiss described how mechanical pressure applied during dental extractions impacted the TMJ [15] and Costen reported that TMD symptoms likely resulted from missing posterior teeth [16]. This marked the beginning of an etiological philosophy that TMD was a consequence of malocclusion, contributing to the belief that occlusal therapy was the primary treatment for TMD. Some practitioners still adhere to this viewpoint, despite the more contemporary, evidence-based reversible and multifactorial approach.

This misconception was further reinforced throughout the 50’s and 60’s. In 1959, dental and occlusal discrepancies were described as the causative factor in TMD implying that addressing these discrepancies would reduce pain, trismus, and swelling [17]. By 1966, TMJ dysfunction was noted as a potential cause of head pain, yet 90% was attributed to occlusal discrepancies [18]. These studies propagated information in medical and dental literature despite numerous reports indicating that occlusal factors were not the sole cause of TMD [19, 20, 21, 22, 23, 24]. This focus contributes to an overemphasis on the identification and treatment of occlusal factors, even though TMD is widely recognized as a multifactorial disorder. It prompts consideration of why the occlusal etiology has persisted and continues to be reinforced despite a longstanding recognition of TMD as a complex disorder with multiple causative factors.

Concurrently, other reports explored a broader and more comprehensive view of TMD. As early as 1949, the Journal of the American Dental Association acknowledged the complexity of TMD, highlighting multiple etiologies and emphasizing the multifactorial understanding of these disorders [25]. A multifactorial philosophy of TMD remains relevant and is widely accepted today. In the mid-twentieth century, potential involvement of psychosocial factors in muscular-related TMD was proposed [26, 27], laying the framework for a biopsychosocial model of TMD. Previous biomedical approaches to chronic pain often resulted in poor patient outcomes and iatrogenic procedures. Consequently, education in the biopsychosocial approach to chronic pain is now recommended to enhance patient improvement [28]. The biopsychosocial framework offers a comprehensive approach to chronic pain disorders, including TMD. The biopsychosocial model is described as an interplay between disease and illness, where the sensory and subjective experience of pain arises from the interactions of biological, psychological, and social factors [29]. Maixner *et al*. [30], demonstrated this in OFP in 2012, as they reported on findings from Orofacial Pain: Prospective Evaluation and Risk Assessment (OPPERA) that supported TMD as a complex disorder best explained by a biopsychosocial model. This model was well-suited to TMD, as these are multisystem problems with overlapping comorbidities resulting in various physical manifestations, behavioral changes, and central nociplastic changes [30, 31].

### 2.2 The evolution of standardized diagnostic criteria

As the understanding of TMD has evolved over time, so have the diagnostic criteria. In 1950, Markowitz and Gerry, outlined basic classifications of intra-articular disorders of the TMJ and used the non-specific term “temporomandibular joint disease” to describe these diagnoses [32]. In 1982, the current term temporomandibular disorders (TMD) was recommended to take the place of broad and non-specific terms such as “temporomandibular joint dysfunction” [13]. However, this terminology continued to fuel confusion as it was an umbrella term for a multitude of disorders of diverse causes.

The first standardized diagnostic criteria were published in 1992. The Research Diagnostic Criteria for TMD (RDC/TMD) was developed to improve consistency in research terminology and diagnosis [33]. Based on the biopsychosocial model, it delineated between muscular and intra-articular TMD, allowing for multiple diagnoses in one individual [33]. While the RDC/TMD exhibited validity in research settings it did not provide clinical guidelines. The Diagnostic Criteria for TMD (DC/TMD) was subsequently published in 2010, as standardized, valid criteria for both clinical and research applications. There were eleven included diagnoses based on the most valid and current evidence [34]. However, limitations included the lack of non-TMD OFP disorders. In 2020, the International Classification of Orofacial Pain (ICOP) developed a set of diagnostic criteria that implemented the DC/TMD and International Classification of Headache Disorders (ICHD-3) that could be used as a guideline for all OFP diagnoses [35]. ICOP provided an evidence-based strategy for describing specific diagnostic criteria based on pathophysiology of a disease process instead of broad, non-specific terms.

### 2.3 The history of predoctoral TMD curriculum

The implementation of structured TMD-related curriculum in predoctoral dental education has faced delays which mirrors the evolving understanding of TMD etiology and diagnostic criteria. The first publication that discussed TMD-related education was published in 1973 in the Journal of Dental Education [36]. A survey of 45 dental schools examined how TMD was addressed in their curricula and teaching approaches to the material, revealing inconsistent and fragmented teaching methods, a lack of standardized content, and limited curriculum time dedicated to TMD. At the time, most schools taught TMD within occlusion courses, with only a few discussing it in the context of behavioral sciences. This likely reflected the ongoing philosophical divide in the field and was potentially related to the early assumptions in the 50’s and 60’s of occlusal and dental causes of TMD. It was concluded that dedicating a specific course to TMD would provide an appropriate framework for teaching about these complex disorders [36]. Even as the clinical approach became more evidently biopsychosocial in nature, the dental field was slow to implement that. In 1992, Solberg and Fricton expressed concern that dental education prioritized technical skills over clinical judgment and devoted more time to acute than chronic pain [37]. They observed a slow integration of chronic pain management into dental curricula and recommended increasing exposure to TMD fundamentals through high-quality interdisciplinary programs [37].

Although the first comprehensive curriculum guidelines were published in 1992 [38], the skewed perception that occlusion was the primary cause of TMD continued to pervade. TMD education became a scapegoat for poor educational outcomes in occlusion as some attributed an expanded focus on TMD in dental education to a diminishing emphasis on occlusion [20]. This remained a struggle throughout the course of the new millennium. Even though experts in the field have repeatedly emphasized the crucial need for education in TMD, gaps have persisted in this discipline. In 2005, Klasser and Green performed a survey of all 56 US and Canadian dental schools to determine if improvements had been made since 1973. This survey pointed to significant improvement in TMD curriculum over 30 years. Notably, this reflected strides within the field of TMD, including the replacement of occlusal etiology with the biopsychosocial model, advances in diagnostic and treatment standards, and advances in imaging. This offered a better scientific foundation for schools to incorporate into their curriculum. The results varied between schools, with only three schools having evidence of an ideal teaching situation, some schools lacking an adequate approach to TMD, and some schools remaining dedicated to outdated concepts. In 2005, the majority of schools reported teaching history taking and comprehensive examinations. However, 58% of them continued to teach an occlusal evaluation as part of a diagnostic work up and less than half included reversible management modalities, such as physical therapy, behavioral management, or injection-based therapies [39]. Fragmentation of material across the dental curriculum remained a challenge, with systematic organization of TMD in the curriculum lacking and primary responsibility of teaching remaining in prosthodontic/restorative and oral surgery departments [39]. They urged changes to educational institutions, such as the American Dental Education Association [39], and not until 2023 were these requests reflected in standards for dental education.

Segmentation of TMD throughout dental school curricula continued to be a sore point as the lack of a systematic approach resulted in poorer educational outcomes [39]. In 2012, Lee *et al*. [22], found that TMD was taught in a standalone course in only one third of dental schools and was more often incorporated into restorative or prosthodontic courses. Some schools reported training TMD in oral medicine or oral surgery departments [22]. This departmental division of content could influence biases in TMD education. For instance, prosthodontics may adopt a more occlusion-focused curriculum, while oral surgery might emphasize a procedural approach. It is notable that schools with dedicated postgraduate training programs in OFP integrated contemporary and evidence-based approaches to predoctoral education, likely due to their interest in this area [39]. With 78 accredited dental schools [40] and 13 postgraduate training programs in the US and Canada [41] only 16% of dental schools have access to this type of programming. It is not a realistic strategy to rely on these programs for implementation of predoctoral content, therefore a separate, structured approach to TMD in the predoctoral curriculum is needed.

Most recently, in 2025, Sangalli *et al*. [42] published the results of a survey regarding predoctoral dental school programs to evaluate TMD curriculum since the inclusion of TMD in The Commission on Dental Accreditation (CODA) standards. Notable improvements since 2005 included 76% of TMD curriculum delivered by OFP specialists. This shift is critical as the individuals teaching TMD to the next generation are those that have specialty training and are most qualified to implement currently accepted standards, such as the biopsychosocial model of TMD etiology and reversible treatment modalities. Possible concerns with non-specialist faculty includes relying on outdated beliefs, such as emphasizing malocclusion in TMD curriculum. Incorporating clinical exposure to TMD remained a challenge for dental schools. However, compared to 2005 when 34% of schools offered no clinical exposure, only 9% of the programs had no student clinical exposure to patients with TMD [42]. Overall, they concluded that TMD instruction varied widely across different dental school programs, but that positive changes were found and markedly improved since the initial reports of TMD curriculum were published in 1973.

## 3. Present

### 3.1 The current state of predoctoral TMD curriculum

Standards for TMD were formalized in 2023 by CODA when predoctoral requirements were addended to include, “graduates must be competent in providing oral health care within the scope of general dentistry, as defined by the school, including: temporomandibular disorders”, without further detail [43]. While this discusses a requirement for competency, it remains the discretion of the individual school to determine how to achieve this, contributing to the lack of standardization that has historically plagued these disorders. Though this narrative review focuses on TMD-related curriculum, it is worth noting that additional limitations of this standard include the lack of other OFP disorders, and a more expansive and standardized approach to both TMD and OFP is critical for future comprehensive predoctoral standards.

The American Academy of Orofacial Pain published comprehensive curriculum recommendations and guidelines for predoctoral education in 2021 that include five domains, including (1) Knowledge Base, (2) Screening, Evaluation, Diagnosis, and Risk Assessment, (3) Health Promotion and Prevention, (4) Clinical Decision-Making, Treatment Planning, Evidence-Based TMD Management, Communication, and Interdisciplinary Collaboration, and (5) Practice Management and Informatics [44]. These core curriculum guidelines provide a current, evidence-based framework for predoctoral TMD education that fulfills the criteria as established by CODA for TMD competency. This can be used as a guide for dental schools to standardize their TMD curriculum in areas where fragmentation and limitations continue to pervade. Fig. 1 provides sample TMD predoctoral curriculum that includes knowledge, skills, and attitudes of dentists upon graduation of their dental school program.

**Fig. 1.** Sample TMD predoctoral curriculum with recommendations of knowledge, skills, and attitudes for graduating dentists. TMD: temporomandibular disorders; TMJ: temporomandibular joint.

### 3.2 Multidisciplinary care and interprofessional education

For chronic pain disorders, including TMD, management strategies focus on improvement in quality of life and function [45]. To achieve this, coordination with a multidisciplinary team is often required. Multidisciplinary management in chronic pain benefits both patients and providers. Patients with chronic pain benefit from accurate diagnosis, appropriate treatment, continuity of care, and improved quality of life, and providers benefit from an increased knowledge network, a reduction in burnout and professional isolation, and decreased medicolegal risk [46, 47]. These benefits are consistent in TMD. In 2024, Matthews and Novosal reported on patient satisfaction results following their care in a multidisciplinary team, with the majority of patients feeling satisfied with their care and preferring to meet with a group of clinicians instead of individually [48]. Interprofessional education (IPE) of healthcare professionals provides the framework to teach students how to work in collaborative environments, preparing them for future practice in multidisciplinary settings in clinical practice. Proposed by the World Health Organization (WHO) in 2010 due to concern for a global healthcare shortage, IPE was a proposed strategy to strengthen health systems at all levels, including education [49]. This disparity is reflected in OFP as only 287 board certified providers exist in the US (Heir, 2024). Given the staggering ratio of 175,000 estimated patients per provider [41], a critical effort of educational initiatives is needed. IPE provides a standard to train students of various health professions together so they may learn effective collaboration to improve health outcomes, thus preparing them for the workplace [41]. IPE initiatives offer a unique solution to the gap in specialists and may improve efforts at providing high quality education-based material to various professionals. Hawkins* et al*. [50] reported on TMD education and training for healthcare professionals due to the current training program shortage. Learners from diverse backgrounds, such as dentistry, medicine, physician assistant, nursing, and physical therapy showed improved knowledge and competency. Most importantly, they felt able to apply what they learned to clinical practice [49]. Core competencies of IPE (Fig. 2) focus on students from multiple disciplines learning from each other and include values and ethics, roles and responsibilities, communication, and teams & teamwork [51]. The aim is not to focus solely on didactic knowledge and individual skills, but to develop professionals better equipped to meet the demands of healthcare needs in a collaborative practice. TMD clinical scenarios are ideal for integrating IPE experiences, as effective patient management includes other comorbidities that often require collaboration among various stakeholders, including dentists, OFP specialists, physicians, physical therapists, and mental health professionals.

**Fig. 2.** Interprofessional education core competencies.

## 4. Future

### 4.1 Live patient experiences

TMD-related education in dental schools needs greater standardization to better equip future clinicians for accurate diagnosis and effective management, and there are unique opportunities in the current environment, summarized in Fig. 3. Strengthening didactic instruction and clinical exposure is crucial for enhancing students’ diagnostic proficiency and preparedness for management of TMD conditions [52]. A three-year retrospective study at a US dental school highlighted this issue, revealing that among the 21,352 patients treated by predoctoral students, only 0.26% of those presenting with symptoms or diagnoses of TMD/OFP received treatment. This suggests that students may struggle with identifying and managing these conditions effectively [53].

**Fig. 3.** Future opportunities in TMD predoctoral curriculum. TMD: temporomandibular disorders; AI: artificial intelligence.

OFP involves a complex interplay of musculoskeletal, neuropathic, and psychosocial factors that cannot be fully grasped through traditional lectures alone. However, significant variability exists in how these concepts are taught to predoctoral students, leading to inconsistencies in their clinical training [52]. Many dental students graduate without sufficient hands-on experience, which affects their confidence and clinical competence [52]. A survey assessing dental students’ perceived competence in OFP management found that third- and fourth-year students felt the least prepared to handle TMD and neuropathic pain conditions. This underscores the need for additional clinical exposure to bridge the gap between theoretical knowledge and real-world application [54]. Despite this, the majority of training experiences in TMD mainly consist of didactic lectures [55]. Without practical experience, students may struggle with key aspects of TMD diagnosis, such as differentiating between musculoskeletal, neuropathic, and referred pain conditions. Integrating live patient experiences provide opportunities for students to apply knowledge in clinical settings, refine decision-making skills, and develop confidence in treating patients with chronic pain conditions.

Standardized patient simulations and virtual patient interactions offer additional pathways to enhance clinical competence, confidence, and preparedness among predoctoral students. Empirical evidence supports the efficacy of such integration. A study on early experiential learning in predoctoral dental education found that when students combined early patient care with biomedical science instruction, they achieved clinical competence approximately 608 hours earlier than peers in traditional programs [56]. Additionally, a 2025 study of dental students reported that personal TMD experiences correlated positively with their knowledge, skills, attitudes, and beliefs about managing TMD patients [57]. This highlights the impact of hands-on learning in accelerating clinical skill development. Furthermore, standardized patient simulations, where trained individuals role-play clinical scenarios, enhance students’ ability to manage patient interactions and prepare for real-world practice. A study on long-term communication retention found that students who participated in a second-year patient simulation more consistently addressed key patient care elements—including cost, treatment risks and benefits, prognosis, and procedural details—than those without this training [58].

Virtual patients (VPs) are also gaining recognition as valuable educational tools. A survey of US and Canadian dental schools found that 63% had integrated VPs, with 90.5% rating them as important or very important for training [59]. VPs offer a structured environment for practicing patient interviews, medical history assessments, and treatment planning, while interactive audio and video features enhance realism, making them a strong complement to live patient interactions. Incorporating multimedia resources, such as instructional videos, further enhances students’ clinical readiness. Watching DC/TMD instructional videos one week before an Objective Structured Clinical Examination (OSCE) significantly boosted students’ confidence in TMD examinations, with participants finding the exam easier, which supported video integration in dental education [60].

### 4.2 Applications of artificial intelligence

The integration of AI in TMD-related education holds significant potential to enhance diagnostic accuracy, improve clinical decision-making, and streamline educational frameworks for dental professionals. AI-based algorithms have proven crucial in diagnosing and predicting outcomes for TMD, conditions known for their multifactorial and complex nature [61, 62, 63, 64]. AI technologies, including machine learning and deep learning, have shown promise in accurately identifying TMJ pathologies, such as joint degeneration and inflammation, which are integral to TMD diagnosis [62, 64, 65]. These systems process a diverse array of data, including imaging, clinical symptoms, and genetic markers, enabling practitioners to diagnose and predict the most effective treatments for each TMD patient with greater precision while reducing human error.

While not a replacement for traditional learning environments or live patient experiences, AI has the potential to enhance TMD-related dental education by offering personalized learning experiences and enhancing diagnostic skills. AI models, such as artificial neural networks, can mimic human brain processing by using interconnected layers of neurons to analyze data, recognize patterns, and make predictions, demonstrating high diagnostic accuracy in differentiating various TMD conditions [63]. AI-driven diagnostic tools can also analyze radiographic images to detect subtle changes in TMJ structures that might be missed by human clinicians [61, 65]. Integrating AI tools into dental education can enable students to gain hands-on experience with real-world applications of AI in TMD diagnosis, ultimately improving clinical decision-making by reducing diagnostic uncertainty and enhancing the identification of complex pain disorders.

Furthermore, incorporating AI into educational platforms can simulate complex clinical scenarios, allowing students to engage in interactive learning experiences. AI-driven virtual patients can present a variety of TMD cases, providing students with a controlled, risk-free environment to practice diagnostic and treatment planning skills. This has a particular promise in environments where institutions do not have dedicated OFP faculty or enough patient volume to provide a standardized experience to every predoctoral dental student. This approach reinforces theoretical knowledge while promoting critical thinking and decision-making, essential for clinical practice. Moreover, AI can analyze individual student performance, pinpointing strengths and areas needing improvement. By offering personalized feedback, AI systems can tailor educational content to address specific learning gaps, creating a more efficient and targeted learning experience. This individualized approach is particularly valuable in complex fields like TMD, where traditional teaching methods may not fully accommodate the limited clinical experiences and the diverse learning needs of students.

### 4.3 Integrating pediatric TMD into predoctoral curriculum

While TMD is often perceived as a condition primarily affecting adults, evidence shows that these disorders can also significantly impact pediatric and adolescent populations [66]. However, traditional dental curricula primarily focus on adult TMD, with limited emphasis on pediatric cases. A recent study of pediatric dentists found low self-perceived knowledge and confidence in diagnosing, screening, and managing TMD in children and adolescents, regardless of experience level [67]. An overwhelming majority (81.6%) indicated a need for continuing education courses and training focused on the management of TMD in pediatric patients [67]. These findings highlight significant gaps in the education and practice patterns of pediatric dentists concerning TMD, underscoring the need for enhanced training and resources in this area.

Untreated pediatric TMD can lead to long-term functional impairments and an increased risk of chronic pain. Integrating pediatric TMD education into predoctoral curricula can facilitate early diagnosis and intervention, potentially preventing these conditions from persisting into adulthood [68]. Pediatric pain management requires unique considerations, as the assessment of pain and the communication of treatment options must be tailored to younger patients. Students exposed to these issues early on can develop the empathy and communication skills needed to address the concerns of both pediatric patients and their families [69]. Incorporating pediatric TMD into the predoctoral curriculum is therefore critical for developing well-rounded dental practitioners capable of addressing diverse patient needs.

### 4.4 Chronic overlapping pain conditions

The multifactorial nature of TMD presents a significant clinical and educational challenge, particularly as it relates to segmenting TMD from OFP as a broader topic in predoctoral education. Given the multifactorial etiology of TMDs, teaching them in a larger OFP curriculum may provide improved foundational didactic knowledge, such as anatomy, neurophysiology, psychosocial influences, and broader concepts of chronic pain such as nociplasticity. Klasser and Greene demonstrated that US and Canadian schools with postgraduate training had dedicated evidence-based curriculum and faculty, supporting the argument that embedded experts teaching the curriculum can improve training of predoctoral students [39].

Inconsistencies in current diagnostic and treatment practices are largely due to the subjective nature of TMD symptoms and the frequent presence of comorbidities, such as chronic overlapping pain conditions (COPCs). COPCs refer to coexisting pain condition that feature multifactorial etiologies and diverse clinical manifestations with a variety of risk factors [70]. Despite their diverse origins, these conditions share symptoms like widespread pain, common risk factors, and pain amplification [70]. These ten painful conditions exhibit higher rates of co-occurrence, and include diagnoses such as fibromyalgia, irritable bowel syndrome, migraine headaches, chronic low back pain, and TMDs [71].

Crucially, the nature of COPCs underscores the importance of individualized care, particularly for TMD, where management response is highly variable. TMDs likely encompass various phenotypes and exist on a continuum, with some individuals experiencing central pain amplification and nociplastic pain, while others do not [72]. Notably, adolescents with TMD or other COPCs are more likely to develop nociplastic pain which can persist as a lifelong issue [73]. Thus, considering central pain states in TMD management is vital. Evaluating where patients fall on this continuum can provide insights into whether treatment should focus more on peripheral or central mechanisms. Using validated questionnaires for appropriate screening [70, 74] is a practical approach that can be incorporated into predoctoral education and seamlessly integrated into dental assessments. Dentists can play a critical role in identifying individuals at risk of developing nociplastic pain, potentially preventing a lifetime of chronic pain and suffering.

### 4.5 Barriers to improving TMD-related education

The improvement of TMD education faces several barriers that hinder progress both academically and clinically. A major barrier is the lack of a standardized curriculum across dental programs. Most institutions lack a uniform approach, with faculty possessing varying levels of expertise in teaching TMD and chronic pain management, as well as limited specialized resources, leading to an inconsistent clinical training environment [75]. It is, therefore, a concern that predoctoral students will learn about TMD from faculty without adequate training or background in TMD and focus on a dental approach to assessment and management, such as occlusion. These inconsistencies create significant disparities in educational quality and depth, leaving many newly trained professionals underprepared to manage TMD effectively [75]. The absence of universally accepted diagnostic criteria for OFP further contributes to confusion and variability in clinical practice. While the DC/TMD system, established by the International Association for the Study of Pain and ICOP, has improved diagnostic standardization, its inconsistent application among practitioners leads to misdiagnoses and suboptimal treatments [75]. This lack of diagnostic consistency complicates the development of educational programs that rely on clear, evidence-based criteria.

Despite profound evidence that TMD is best understood through a biopsychosocial framework, challenges exist in implementing these concepts in clinical and educational settings. These disorders lag behind their medical counterparts in the implementation of this framework. In 2019, Sharma *et al*. [76] identified five main barriers to integrating the biopsychosocial framework into diagnosis and management of OFP, including culture and society, healthcare settings, health services, instruments and constructs, and health literacy and education. This may explain why inaccurate and outdated pathophysiology of TMD is still promoted and remnants of occlusal etiologies remain in dental education. Training in the biopsychosocial model of pain improves knowledge and attitudes about pain, and supports guideline adherence [28]. Therefore, incorporating the biopsychosocial framework into the dental curriculum would improve dental student TMD-related knowledge.

The exclusive focus on TMD as the sole standard in the predoctoral dental curriculum presents a significant barrier. This narrow approach overlooks the broader spectrum of OFP disorders, leaving a substantial gap in dental education. Without a comprehensive approach to OFP disorders beyond TMD, students miss critical training necessary for addressing the full range of OFP conditions. The multifactorial nature of OFP adds another layer of complexity. The pathophysiology of these conditions involves complex interactions between nociceptive, inflammatory, and neuroplastic mechanisms, which complicates the development of educational frameworks that can be easily understood by clinicians without specialized training in neurobiology or pain science [77]. Another critical barrier is the inadequate funding for research on TMD and OFP, which restricts the availability of evidence-based educational resources. While there is a growing body of literature, the funding allocated to TMD and OFP research remains disproportionate when compared to other chronic pain conditions, such as low back pain. According to the National Institutes of Health (NIH) database, research funding for TMD was significantly lower than for other pain-related conditions, limiting the scope of new educational materials and guidelines based on recent findings [78].

## 5. Limitations

Though this narrative review provides a perspective on the climate of predoctoral education, it is not a systematic review and therefore relies on expert opinion. Additionally, the focus on US and Canadian schools limits the perspective to one geographic location, and does not include a larger global context.

## 6. Conclusions

Although this past, present, and future perspective focused on US and Canadian contexts, strengthening TMD-related education globally is essential for reducing patient suffering associated with these disorders, improving patient outcomes, and supporting clinician confidence. To enhance the education of future dental professionals, it is crucial to restructure the predoctoral curriculum to include dedicated courses on TMD. The current narrow focus on TMD fails to prepare students for the diverse challenges they will face in practice, particularly given the multifactorial nature of these disorders. Future directions include expanding the curriculum to encompass a variety of OFP conditions to ensure a more comprehensive educational experience. These courses should be taught by faculty with recognized credentials and substantial experience in TMD, ensuring that students benefit from expert knowledge and practical insights. Furthermore, promoting hands-on clinical experiences is essential; real-world exposure will help students bridge the gap between theoretical knowledge and clinical application, enhancing their confidence and competence in diagnosis and management.

Integrating these elements into dental education will equip students with the skills necessary for effective patient care. By adopting a more holistic and standardized approach to TMD education, dental schools can better prepare graduates to navigate the complexities of orofacial pain, ultimately leading to a higher standard of care and patient satisfaction. Emphasizing interprofessional collaboration and the latest diagnostic and treatment technologies will ensure that future practitioners are poised to meet the evolving needs of their patients.
